# Acute and chronic effects of aerobic exercise on blood pressure in resistant hypertension: study protocol for a randomized controlled trial

**DOI:** 10.1186/s13063-017-1985-5

**Published:** 2017-06-02

**Authors:** LS Nascimento, AC Santos, JMS Lucena, LGO Silva, AEM Almeida, MS Brasileiro-Santos

**Affiliations:** 1 Laboratório de Estudos do Treinamento Físico Aplicado a Saúde, Departamento de Educação Física, Universidade Federal da Paraíba, Castelo Branco I, CEP 58051-900 João Pessoa, Paraíba Brasil; 2 Programa Associado de Pós-Graduação em Educação Física UPE/UFPB, João Pessoa, Paraíba Brasil; 3 Universidade Federal do Tocantins, Campus Universitário de Tocantinópolis, Centro, CEP 77900-000 Tocantinópolis, Tocantins Brasil

**Keywords:** Hypertension, Exercise, Post-exercise hypotension, Autonomic nervous system, Hemodynamics

## Abstract

**Background:**

Resistant hypertension is a specific condition that affects approximately 10% of subjects with hypertension, and is characterized by persistently high blood pressure levels even using therapy of three or more antihypertensive agents or with blood pressure control using therapy with four or more antihypertensive agents. Changes in lifestyle, such as physical exercise, are indicated for controlling blood pressure. However, investigating studies about this therapy in individuals with resistant hypertension are few.

**Methods/design:**

This is a randomized controlled clinical trial. Forty-eight patients with resistant hypertension will be submitted to perform four short-term interventions: aerobic exercise sessions (mild-, moderate- and high-intensity) and control session, in random order and on separate days. After the short-term sessions, the patients will be randomly allocated into four groups for 8 weeks of follow-up: mild-, moderate- and high-intensity aerobic exercise, and a control group. The primary outcome is the occurrence of blood pressure reduction (office and ambulatory analysis, and acute and chronic effects). Secondary outcomes are autonomic and hemodynamic mechanisms: cardiac and vasomotor autonomic modulation, spontaneous baroreflex sensitivity, forearm blood flow and vascular resistance.

**Discussion:**

The importance of exercise for hypertension has been known for decades, but little is known about the effects on patients with resistant hypertension. This study will help to understand whether different aerobic exercise intensities can induce different responses, as well as by what mechanisms adjustments in blood pressure levels may occur.

**Trial registration:**

ClinicalTrials.gov, ID: NCT02670681. Registered on 28 January 2016 (first version); Brazilian Registry Platform Clinical Trials: protocol RBR-5q24zh. Registered on 24 June 2015.

**Electronic supplementary material:**

The online version of this article (doi:10.1186/s13063-017-1985-5) contains supplementary material, which is available to authorized users.

## Background

Systemic hypertension is a multifactorial clinical condition characterized by persistently high blood pressure (BP) levels with systolic BP (SBP) ≥140 mmHg and/or diastolic BP (DBP) ≥90 mmHg [1]. In a particular condition, hypertension can be classified as resistant hypertension, which is characterized by persistently high BP levels even with therapy involving three or more antihypertensive agents in appropriate doses and combinations, or controlled BP in therapy with four or more antihypertensive agents [2], as well as failure to maintain BP levels below 140/90 mmHg, even with the appropriate use of three or more antihypertensive agents and at least one diuretic being necessary [3]. The prevalence of resistant hypertension is highly variable depending on the evaluated population, but approximately 10% of the hypertensive population has this resistance [1].

Given the lack of pharmacological responsiveness of this population, nonpharmacological strategies are encouraged. These include restricting salt intake, reducing alcohol consumption, stopping smoking, losing weight, changing diet and doing regular physical activity [1]. The importance of physical exercise in promoting a reduction in resting BP levels is well established in the academic literature, and is a phenomenon known as post-exercise hypotension (PEH) [4]. PEH could occur due to alterations in diverse mechanisms such as reduced peripheral vascular resistance and/or cardiac output [5–7], increased vasodilator bioavailability [6, 8, 9], reduced sympathetic nerve activity, increased parasympathetic modulation and improved baroreflex sensitivity [10, 11].

Previous studies have found decreased BP levels after a single aerobic exercise session for hypertensive patients [12–21]. There is a wide range of magnitude (between −2 mmHg and −12 mmHg) and duration (between 4 and 16 h) of PEH, which is probably due to individual characteristics and different aerobic exercise protocols (e.g., intensity and duration) [22]. BP reduction is also found in follow-up studies [23–27]. In a meta-analysis of normotensive and hypertensive patients, Fagard [28] showed a 3.3-mmHg decrease in SBP, and 3.5 mmHg for DBP. Such reductions in chronic levels are often more discreet, but very important clinically, where a 2-mmHg reduction may decrease the risk of myocardial infarction by about 6%, and the risk of developing coronary artery disease by 4% [3].

For subjects with resistant hypertension, there are few studies involving physical exercise. Clinical trials [29–32] have identified significant reductions in SBP and DBP levels after 12 weeks of continuous moderate aerobic exercise training (based on lactate concentration) and strength training (resistance exercise in a pool). Recently, a crossover trial investigated short-term exercise (mild and moderate intensity) effects in resistant hypertension and found significant reductions in both SBP and DBP levels at both intensities [33]. These studies show the importance of physical exercise as a therapeutic strategy for this population. However, it is still unclear which physical exercise characteristics can result in better outcomes in follow-up studies for patients with resistant hypertension, as well as the possible mechanisms linked to adjustments in BP levels. Exercise characteristics, such as intensity, duration, frequency and type, are associated with different BP responses. Aerobic exercise intensity is a characteristic that seems to influence the magnitude and duration of PEH, but there is no consensus about the magnitude of hypotension and/or the intensity which is more effective in reducing BP levels. Eicher et al. [34] reported that high-intensity interval-training exercise promotes PEH; however, other authors found that lower intensities have been more effective in reducing BP levels [35]. Therefore, studies are inconclusive in presenting the most effective intensity or identifying differences in hypotensive responses to varying intensities used in clinical trials [17, 25, 36–39].

Thus, a trial testing the efficacy of different intensities of aerobic exercise to control BP is necessary and important. Considering the recent findings on physical exercise in resistant hypertension and different responses to exercise intensities, it is hypothesized that short-term sessions of aerobic exercise of mild, moderate and high intensities can promote PEH as a primary outcome; furthermore, follow-up aerobic exercise training for 8 weeks can reduce the BP levels. Additionally, adjustments are expected in the autonomic and hemodynamic variables, such as reduced sympathetic modulation, increased parasympathetic modulation, improved baroreflex sensitivity, decreased forearm vascular resistance, and a consequent increase in blood flow.

## Rationale

It is known that hemodynamic, humoral and neural changes are associated with hypertension. Pharmacological therapy affects these variables and helps to control BP. However, some subjects with hypertension are nonrespondent to this therapy, and are classified as resistant hypertensive patients. Acute and chronic aerobic exercise reduces BP, thereby adjusting autonomic and hemodynamics, improving parasympathetic modulation, baroreflex sensitivity, vasodilator response and attenuates sympathetic modulation and vascular resistance. Different aerobic exercise intensities could stimulate diverse physiological mechanism responses, and, therefore should produce different adjustments in BP. Even when not responding properly to drug strategies that act on different variables depending on antihypertensive class, it is possible that simultaneous autonomic and hemodynamic adjustments caused by physical exercise promote reduced BP.

## Research question

Is there reduced BP after different short-term aerobic exercise intensities in resistant hypertensive patients? Could reduced BP persist after aerobic training?

What mechanisms (autonomic and hemodynamic) could be responsible for PEH in these resistant hypertensive patients after short-term and long-term aerobic exercise at different intensities?

## Methods/design

**Fig. 1:**
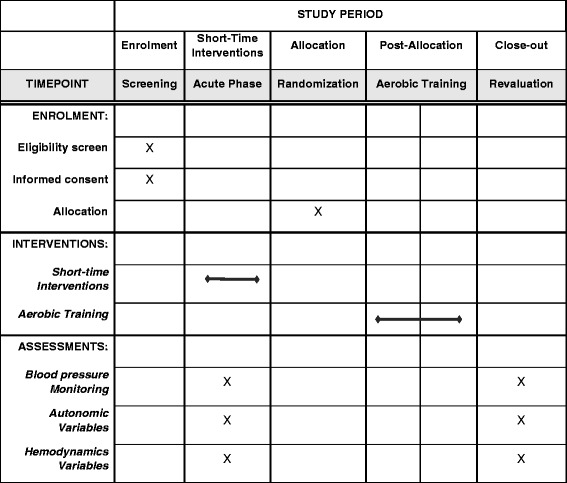
Standard Protocol Items: Recommendations for Interventional Trials (SPIRIT) schedule of enrollment, interventions and assessments

**Fig. 2:**
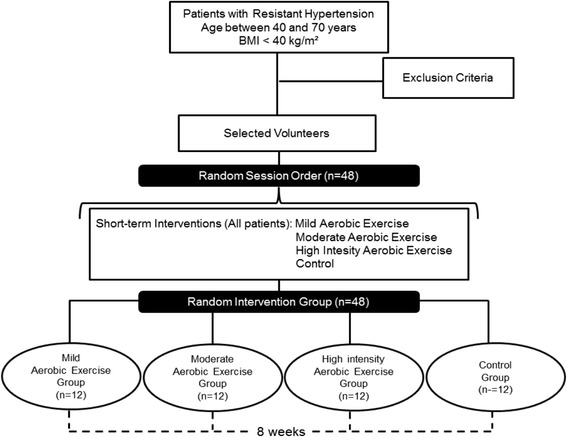
Flow chart of selection and interventions. BMI: Body Mass Index. kg: kilogram. m: meter

This is a randomized controlled clinical trial with single-blind data analysis. The Standard Protocol Items: Recommendations for Interventional Trials (SPIRIT) flow chart and enrollment schedule, interventions and assessments for the trial are given in Fig. 1 and the SPIRIT Checklist in Additional file 1. Figure 2 presents the experimental research design stages. In addition to ongoing medical monitoring, all individuals will be assessed by anamnesis, anthropometric measurements, biochemical tests, echocardiogram, cardiopulmonary exercise test, information about lifestyle (including dietary habits), and habits/history of physical activity before each short-term intervention and after the follow-up period.

### Eligible participant and exclusion criteria

Patients with resistant hypertension, who are aged between 40 and 70 years, will be recruited from the Lauro Wanderley University Hospital at the Federal University of Paraiba, Brazil, being men or women (women must be postmenopausal, nonmenstruating for at least 1 year and not using hormone replacement therapy), with Body Mass Index ˂40 kg/m^2^ and able to do physical exercise. Patients with resistant hypertension when BP levels are ≥140/90 mmHg for SBP/DBP, respectively, using three or more antihypertensive agents in appropriate doses and combinations, or with controlled BP using four or more antihypertensive agents, will be considered [3] in addition to considering ambulatory BP monitoring (ABPM) records. Controlled BP will be considered for SBP and DBP: at 24 h <130 mmHg and <80 mmHg; awake <135 mmHg and <85 mmHg; and sleeping <120 mmHg and <70 mmHg [40].

After clinical assessment and verifying medical records and biochemical data, patients can be excluded for having a history of ischemic stroke, coronary heart disease, chronic obstructive or restrictive pulmonary disease, peripheral arterial disease, hypo/hypernatremia, hyper/hypothyroidism, chronic atrial fibrillation or a change in drug therapy for an experimental protocol. In the follow-up phase, patients who do not participate in 90% of the exercise training sessions, miss three consecutive training sessions or present any osteoarticular disease that prevents them from continuing the physical training program will be considered as sample loss.

### Random allocation sequence

Randomization will be done using a sealed opaque envelope. All the patients will be submitted to perform four short-term interventions (within design): mild aerobic exercise, moderate aerobic exercise and high-intensity aerobic exercise and a control session, in random order and on separate days. After the short-term sessions, all the patients will be randomly allocated into four groups and followed up for 8 weeks (between design): mild aerobic exercise group, moderate aerobic exercise group, high-intensity aerobic exercise group and the control group.

### Acute exercise interventions

Considering the recommendations of the American College of Sports Medicine [41] and adjusting tolerance estimated by untrained resistant hypertensive patients, each aerobic exercise session will be 40 min long (warm-up: 5 min; intervention: 30 min; and cool-down: 5 min). The control session also will last 40 min, but with rest in a seated position.

The cardiopulmonary exercise test will be used to determine the maximal aerobic capacity at peak exercise (VO_2_peak), anaerobic threshold and the respiratory compensation point. The heart rate corresponding to the anaerobic threshold and the respiratory compensation point will be used for the prescription of the mild-, moderate- and high-intensity protocols:

- Mild aerobic exercise: continuous 30-min intervention 10% below the anaerobic threshold to the anaerobic threshold point
- Moderate aerobic exercise: continuous 30-min intervention at the anaerobic threshold point to the respiratory compensation point
- High-intensity aerobic exercise: interval of 30-min intervention: 10 stimuli of 1 min above the respiratory compensation point with 2 min interval of passive rest after each stimulus
- Control: 40 min of rest in a seated position

### Aerobic exercise training

After short-term interventions, the patients will be randomly allocated into four groups for 8 weeks, training three times per week. It will be recommended to the patients to continue with their same daily activities and food. The chronic interventions will be in the same format as the acute interventions:

- Mild aerobic exercise group: 24 sessions of continuous 30-min intervention 10% below the anaerobic threshold to the anaerobic threshold point
- Moderate aerobic exercise group: 24 sessions of continuous 30-min intervention at the anaerobic threshold point to the respiratory compensation point
- High-intensity aerobic exercise group: 24 sessions of 30-min interval intervention: 10 stimuli of 1 min above the respiratory compensation point with 2-min interval of passive rest after each stimulus
- Control group: without aerobic exercise training. Patients will be instructed to not initiate any supervised physical exercise program until the end of the reevaluations

All patients will be frequently informed about the research progress and its partial results throughout the follow-up period. In addition, the researchers will maintain constant contact with the physicians responsible for each patient. The physicians and patients will receive a final report with the results at the end of study.

### Measurements

Individuals will receive the following instructions 24 h prior to measuring their biological signals: maintain their normal drug-use routine and sleep and meal hours; do not drink alcohol, tea, coffee, soda, or any food/drink containing caffeine. In addition, on the experiment day they will be asked to ingest a light meal 2 h before initiating the protocol. Patients will be asked to record their activities 24 h following the experiment, such as working hours, sleep schedule, meals, use of medication and any complications that they consider important (e.g., stressful situations). The same instructions will be given in the revaluation after the follow-up period.

In each short-term experimental session and follow-up phase, the biological BP signal, electrocardiogram (ECG) and blood flow will be continuously measured using 500-Hz frequency per channel in WINDAQ software (DATAQ Instruments DI-720 Acquisition, Akron, OH, USA). BP will be recorded by ABPM (Dynamapa Cardios®, São Paulo, Brazil) measured in the nondominant arm by a Dixtal® oscillometric semiautomatic monitor (Dixtal®, DX 2020, Manaus, Brazil) and by Finometer oscillometric equipment (Ohmeda 2300 Monitoring Systems, Englewood, CO, USA). BP values will be obtained immediately before exercise, 30 and 60 min after each intervention (ABPM, Dixtal® and Finometer methods), and 24 h (ABPM) after the end of the intervention. ECG will be evaluated in the DII derivation (right and left arm). Forearm blood flow will be collected by venous occlusion plethysmography [42] apud [43] (Hokanson®/EC6 plethysmograph, Bellevue, WA, USA). During blood flow signal collection, BP values will be registered by Dixtal® oscillometric semiautomatic monitor (Dixtal®, DX 2020, Manaus, Brazil). All the measurement methods will be repeated in an experimental session without any intervention after the follow-up period in the same sequence and procedure.

## Outcomes

### Primary outcome

#### Blood pressure

For the BP measurement in the office, analysis will consider the difference between post-intervention and pre-intervention values. For ambulatory analysis, BP will be considered as sleep and awake periods, and the difference between 24-h post-intervention and pre-intervention values. In the follow-up analysis, baseline BP values (considering control session values without intervention) and post-training BP values will be evaluated.

### Secondary outcomes

#### Cardiac autonomic modulation

- Linear analysis – time domain (heart rate variability) and frequency domain (absolute and normalized spectral component of low- and high-band frequencies to evaluate sympathetic and parasympathetic modulation, respectively, and autonomic balance) [44]
- Nonlinear analysis – symbolic analysis using four symbolic families (0 V%, 1 V%, 2LV% and 2ULV%) to evaluate sympathetic and parasympathetic modulation and Shannon entropy [45–47]

#### Vasomotor autonomic modulation

- Linear analysis – time domain (BP variability) and frequency domain: low absolute component frequency (sympathetic vasomotor index) [48]

#### Spontaneous baroreflex sensitivity

- Linear analysis – frequency domain method: alpha index by cross-analysis between SBP spectral power and RR interval spectral power (spontaneous baroreflex control of heart rate) [49, 50]

#### Forearm blood flow and peripheral vascular resistance

- Evaluation of blood flow via assessment of the vascular tissue volume change in the forearm. The rate of the volume change is proportional to the rate of arterial inflow [43]. Forearm vascular resistance will be calculated as the ratio of mean BP and forearm blood flow

## Statistical analysis

### Power and sample size

Sample size was calculated using ABPM as the main outcome. Considering the high variance in the results with resistant hypertensive patients [29, 30] and the meta-analysis results of Fagard [28] with hypertensive patients, BP reduction after endurance training of 7 mmHg from SBP and 5 mmHg from DBP was used in the calculation. In order, to provide 80% power to detect a difference of 3 mmHg between the four distinct conditions (exercise intensity and control), a total of 32 patients are indicated to give sufficient power for the study (*n* = 8 for each group). Considering the possible sample loss, 12 patients will be included in each group.

### Analysis plan

Intragroup and intergroup differences between dependent variables (BP, autonomic modulation, baroreflex sensitivity, blood flow and vascular resistance) considering pre and post interventions (short-term), baseline and post intervention (dependent factor), and exercise intensities or control (independent factor) will be analyzed by two-way analysis of variance (ANOVA) for repeated measures with the Bonferroni post hoc test. Multiple linear regression will be used for analyzing confounding factors (dietary habits and unsupervised physical exercise practice in the follow-up period). Pearson’s correlation test will be used for analyzing the relation between short-term and long-term results. Intention-to-treat will be considered for the patients with an incomplete follow-up period. A *p* value < 0.05 will be considered significant for all evaluations.

## Discussion

The prevalence of resistant hypertension is about 10% among hypertensive patients. This number is very representative considering the overall prevalence of hypertension and consequent harm to cardiovascular health often associated with morbidity and mortality. Changes in lifestyle are continually recommended, especially in diet adjustment and regular physical exercise.

The guidelines on evaluating BP levels and resistant hypertension pay particular attention to the possibility of misdiagnosis, difficulty of adherence to a newly started treatment, adequacy of combining antihypertensive classes, adequacy of each drug dosage, as well as cases of white coat hypertension. Therefore, resistant hypertension can be real or only apparently spurious [1]. In the present study, the inclusion of each volunteer will consider medical care, medication time without changing for at least 1 year, and the use of ABPM for an appropriate confirmation of resistant hypertension.

There are only a few studies that have investigated physical exercise as an alternative therapy for resistant hypertension treatment, and they have presented promising results [29, 30, 32, 33]. However, these studies did not perform randomization, allocation blindness, appropriate pairing, or detailed description of methods (mainly statistical analysis). Also, the results are also among the main criticisms of the experts [51]. Therefore, the employed study design could hinder correctly interpreting the data, and consequently limit the scientific evidence on clinical utilization of exercise as a tool for hypertensives.

Considering the acute and chronic potential effects of exercise on BP, the lack of responsiveness to antihypertensive drugs can adjust the mechanisms involved in BP control. It is possible that there is also an unexpected response to exercise, since an adverse response to exercise is not uncommon. A considerable percentage of hypertensive or nonhypertensive patients already had an adverse response in previous studies [52–54]. Thus, resistant hypertensive patients can also have difficulty attaining reduced BP.

Finally, any analysis of BP-level modifications should look for explanations for such adjustments. Previous studies with this population analyzed peripheral [33] or central [29] variables to try to explain the reduced BP. This study aims to deepen these analyzes by simultaneously collecting signals that will be assessed at baseline and post intervention, and to evaluate central and peripheral modulatory variables through cardiovascular autonomic evaluation and vasodilatory response. The acute and chronic benefits of different exercise intensities to be described in this study could provide a new treatment strategy for resistant hypertension which could be included in clinical practice.

## Trial status

The trial is still recruiting patients.

## Additional file

Additional file 1: SPIRIT Fillable Checklist: recommended items to address in clinical trial protocol and related documents. (DOC 120 kb)

## Abbreviations

ANOVA: Analysis of variance
BMI: Body Mass Index
VO_2peak_: Peak oxygen uptake
